# HARNESSING THE POWER OF PROFESSIONAL NETWORKS: IDENTIFYING FACILITATORS AND BARRIERS TO CAREERS IN GEROPSYCHOLOGY

**DOI:** 10.1093/geroni/igz038.035

**Published:** 2019-11-08

**Authors:** Rebecca S Allen, Brian Carpenter

**Affiliations:** 1 The University of Alabama, Tuscaloosa, Alabama, United States; 2 Washington University in St. Louis, St. Louis, Missouri, United States

## Abstract

This symposium presents data from a mixed method study designed to explore how to harness the power of professional networks to increase the pipeline of trainees pursuing careers in academic and clinical geropsychology. Participants were recruited through professional websites, listserves, announcements at annual meetings, and emails from directors of clinical training at pre-and post-doctoral training sites. A total of 107 geropsychologists completed the survey, including 28 graduate students/interns and 76 post-doctoral psychologists ranging from early to late career. The mean age of respondents was 39.18 (SD = 12.05). The sample was largely female (71.7%) and Caucasian (88.7%), paralleling previous work. The first paper describes attractive and unattractive aspects of clinical and academic career options, including gender differences in perceptions of the feasibility of changing career foci. The second paper examines perceptions of clinically-focused and academic jobs, and discrepancies between professional psychologists’ actual and ideal job activities. Examining content analysis of 28 qualitative transcripts, the third paper focuses on VA training and the convenience and comfort of transitioning into a VA job after training within this system, identifying benefits and challenges of work in the VA. The fourth explores these 28 transcripts to identify perceptions of mentorship and supervision during training, including time spent in the training sites (graduate school vs internship) and observations of how working environments impact future career choices. Discussion will address the critical shortage of geropsychologists in academic and clinical settings and strategies to improve the professional pipeline to increase the numbers of trainees pursuing these careers.

